# A phase I/II trial of avelumab combinations with ivuxolimab, utomilumab, and radiation therapy in patients with advanced gastrointestinal malignancies

**DOI:** 10.1093/oncolo/oyaf032

**Published:** 2025-03-26

**Authors:** Jibran Ahmed, Anne Knisely, Carlos Torrado, Bettzy Stephen, Yali Yang, Juhee Song, Anas Alshawa, Abdulrazzak Zarifa, Anuja Jhingran, Eugene J Koay, Van Karlyle Morris, Milind Javle, Robert A Wolff, Funda Meric-Bernstam, Shubham Pant, Jordi Rodon, Aung Naing

**Affiliations:** Department of Investigational Cancer Therapeutics, The University of Texas MD Anderson Cancer Center, Houston, TX 77030, United States; Department of Gynecologic Oncology and Reproductive Medicine, The University of Texas MD Anderson Cancer Center, Houston, TX 77230, United States; Department of Investigational Cancer Therapeutics, The University of Texas MD Anderson Cancer Center, Houston, TX 77030, United States; Department of Investigational Cancer Therapeutics, The University of Texas MD Anderson Cancer Center, Houston, TX 77030, United States; Department of Investigational Cancer Therapeutics, The University of Texas MD Anderson Cancer Center, Houston, TX 77030, United States; Department of Biostatistics, The University of Texas MD Anderson Cancer Center, Houston, TX 77030, United States; Department of Investigational Cancer Therapeutics, The University of Texas MD Anderson Cancer Center, Houston, TX 77030, United States; Department of Investigational Cancer Therapeutics, The University of Texas MD Anderson Cancer Center, Houston, TX 77030, United States; Department of Radiation Oncology, The University of Texas MD Anderson Cancer Center, Houston, TX 77030, United States; Department of Radiation Oncology, The University of Texas MD Anderson Cancer Center, Houston, TX 77030, United States; Department of Gastrointestinal Medical Oncology, The University of Texas MD Anderson Cancer Center, Houston, TX 77030, United States; Department of Gastrointestinal Medical Oncology, The University of Texas MD Anderson Cancer Center, Houston, TX 77030, United States; Department of Gastrointestinal Medical Oncology, The University of Texas MD Anderson Cancer Center, Houston, TX 77030, United States; Department of Investigational Cancer Therapeutics, The University of Texas MD Anderson Cancer Center, Houston, TX 77030, United States; Department of Gastrointestinal Medical Oncology, The University of Texas MD Anderson Cancer Center, Houston, TX 77030, United States; Department of Investigational Cancer Therapeutics, The University of Texas MD Anderson Cancer Center, Houston, TX 77030, United States; Department of Investigational Cancer Therapeutics, The University of Texas MD Anderson Cancer Center, Houston, TX 77030, United States

**Keywords:** avelumab, ivuxolimab, utomilumab, PD-L1, OX40, 4-IBB, immunotherapy, pancreatic, gastric, liver, colorectal

## Abstract

**Background:**

Checkpoint agonists utomilumab (4-1BB agonist) and ivuxolimab (OX40 agonist) enhance T_effector_ cell function. Preclinical studies suggest that combining these drugs with avelumab (anti-PD-L1 antibody) can potentially synergize this effect. In addition, tissue abscopal effects of radiation therapy may improve antigen presentation, complementing PD-L1 blockade. We conducted a single institution, open-label, multi-arm, non-randomized, phase 1/2 clinical trial of avelumab in combination with ivuxolimab, with or without utomilumab, and radiation therapy in patients with advanced solid tumors. Herein, we present a subgroup analysis in patients with gastrointestinal (GI) tumors (pancreatic, colon, gastric, and hepatocellular).

**Methods:**

The primary objectives of this study were to assess safety, tolerability, and dose-limiting toxicities. The secondary objectives were to evaluate efficacy including response rate, progression free survival (PFS), as determined by immune-related Response Criteria in Solid Tumors (irRECIST) and overall survival (OS).

**Results:**

Thirty-one patients with pancreatic (*n* = 21), colorectal (*n* = 8), hepatocellular (*n* = 1), and gastric (*n* = 1) cancers were included in this study. The most common treatment-related adverse events (TRAEs) were chills (13%), diarrhea (10%), colitis (10%), fatigue (6%), and fever (6%). There were 3 instances of grade 3 diarrhea and colitis (10%) without any other grade ≥ 3 TRAEs Among the 24 patients evaluable for response, 9 (37.5%) had immune-related stable disease (irSD) and 14 (58.3%) had immune-related progressive disease (irPD). One patient had clinical progression without radiological confirmation. The median PFS was 2 months. Median OS was 5.6 months.

**Conclusion:**

Combining avelumab with co-stimulatory checkpoint agonists produces modest activity without added safety concerns in patients with advanced GI malignancies (ClinicalTrials.gov Identifier: NCT03217747).

**Lessons learned**

- This study on the combination of utomilumab, ivuxolimab, anti-PD-L1, and radiation therapy in humans demonstrates that the combination is safe. The findings suggest avenues for future research to optimize immunotherapy combinations, potentially enhancing the efficacy observed in this clinical trial.

## Discussion

The safety profile of this combination of co-inhibitory and co-stimulatory immunotherapies revealed mainly grade 1-2 adverse events (AEs), with no grade 4 or 5 AEs linked to the study drugs. Notably, 9.7% of patients experienced grade 3 diarrhea and colitis.

The clinical benefit was limited, with only 37.5% of patients achieving an immune-related stable disease (irSD) response according to irRECIST. Of these, 8 patients were in treatment arms B and C, which used immunotherapy without radiation, making it difficult to evaluate the impact of radiation therapy due to the small sample size in arms E and F. Prior research had shown limited efficacy of single-agent anti-PD-L1 therapy in pancreatic and colorectal cancers.

Preclinical studies suggested that combining co-stimulatory checkpoint agonists with checkpoint inhibitors could synergistically control tumor growth and enhance T-cell responses. However, this trial did not observe meaningful improvements with the addition of OX40 and 4-1BB agonists. A previous phase 1 trial with ivuxolimab (OX40) and utomilumab (4-1BB) showed better outcomes in non-GI cancers, which may be more responsive to immunotherapy.

The study also explored sequential treatment, first stimulating T cells with OX40 agonist ivuxolimab and then using avelumab, based on preclinical mouse models showing additive effects. However, changes in dosing during the trial limited definitive conclusions.

The study faced limitations due to the heterogeneous GI malignancy cohort, small sample sizes, and changes in dosing. Ongoing research aims to understand the immunophenotype and correlates with this combination.

## Trial Information

**Table:** 

	
Disease	Gastrointestinal cancers
Stage of disease/ treatment	Advanced or metastatic
Prior therapy	Subjects with prior anti-PD-1, anti-PD-L1 treatment were excluded. For Arms A and D, subjects may not have had prior 4-1BB treatment. For Arm B, subjects may not have had prior OX40 treatment. For Arm C, subjects may not have had prior 4-1BB or OX40 treatment
Type of study	Phase I/II
Primary endpoint	Safety and tolerability assessed by monitoring the frequency, duration, and severity of adverse events (AEs) according to National Cancer Institute Common Terminology Criteria for Adverse Events (CTCAE) version 4.03. Evaluation of CD8 biomarkers from tumor and blood biospecimens.
Secondary endpoints	Objective response rate determined by radiographic disease assessments per RECIST v1.1 and irRECIST. Progression-free survival, defined as the time from Cycle 1 start date until the earliest date of disease progression, as determined by investigator assessment of objective radiographic disease assessments per RECIST v1.1 and irRECIST, or death due to any cause, if occurring sooner than progression. Duration of response determined by radiographic disease assessment, defined as the time from earliest date of disease response until the earliest date of disease progression per RECIST v1.1 and irRECIST, or death due to any cause, if occurring sooner than progression. Overall survival determined from the Cycle 1 start date until death due to any cause. Response data in irradiated and non-irradiated lesions. Evaluation of various immune biomarkers from tumor and blood biospecimens
Investigator’s analysis	Level of activity did not meet planned end point

## Additional details of endpoints or study design

### Study design

This study was a single-institution, open-label, multi-arm, multi-cohort, non-randomized, phase 1/2 clinical trial of avelumab in combination with ivuxolimab (OX40 agonist), utomilumab (4-1BB agonist), and radiation therapy in patients with advanced malignancies. The study included six arms (A to F), with GI cancer patients enrolled in arms B, C, E, and F (**Table 1**). Patients in arm B received avelumab plus ivuxolimab; Arm C received avelumab plus ivuxolimab and utomilumab; Arm E received avelumab and ivuxolimab plus radiation therapy; and Arm F received avelumab, ivuxolimab, and utomilumab plus radiation therapy. Patients were allocated to the arms based on the slot availability.

**Table 1.** Cohorts and treatment schedules

Cohorts	Treatment	Treatment schedule	Dosing
**Arm B** Expansion	Avelumab (PD-L1 inhibitor) + ivuxolimab (OX40 agonist)	Ivuxolimab: starting Cy1D1 Avelumab: initially started Cy3D1; partway revised to start Cy1D15	Avelumab: 10 mg/kg Ivuxolimab: 0.3 mg/kg Both IV every 2 weeks
**Arm C** Escalation	Avelumab (PD-L1 inhibitor) + ivuxolimab (OX40 agonist) + utomilumab (4-1BB agonist)	Avelumab, ivuxolimab, and utomilumab: starting Cy1D1	Avelumab: 10 mg/kg Ivuxolimab: 0.3 mg/kg Both IV every 2 weeks Utomilumab: 20 mg IV every 4 weeks
**Arm C** Expansion	Avelumab (PD-L1 inhibitor) + ivuxolimab (OX40 agonist) + utomilumab (4-1BB agonist)	Ivuxolimab and utomilumab: starting Cy1D1 Avelumab: initially started Cy3D1; partway revised to start Cy1D15	Avelumab: 10 mg/kg Ivuxolimab: 0.3 mg/kg Both IV every 2 weeks Utomilumab: 20 mg IV every 4 weeks
**Arm E** Escalation Schedule 1 Dose Level 1	Avelumab (PD-L1 inhibitor) + ivuxolimab (OX40 agonist) + XRT	Avelumab andivuxolimab: starting Cy1D1 XRT: starting Cy1D2 (through Cy1D11)	Avelumab: 10 mg/kg Ivuxolimab: 0.3 mg/kg Both IV every 2 weeks XRT: 60 Gy in 10 fractions
**Arm E **Escalation Schedule 2 Dose Level 1	Avelumab (PD-L1 inhibitor) + ivuxolimab (OX40 agonist) + XRT	XRT: starting day − 14 (through day − 1) Ivuxolimab:starting Cy1D1 Avelumab: starting Cy1D15	Avelumab: 10 mg/kg Ivuxolimab: 0.3 mg/kg Both IV every 2 weeks XRT: 60 Gy in 10 fractions
**Arm F** Escalation Schedule 1 Dose Level 1	Avelumab (PD-L1 inhibitor) + ivuxolimab (OX40 agonist) + utomilumab (4-1BB agonist) + XRT	Avelumab, ivuxolimab, and utomilumab: starting Cy1D1 XRT: starting Cy1D2 (through Cy1D11)	Avelumab: 10 mg/kg Ivuxolimab: 0.3 mg/kg Both IV every 2 weeks Utomilumab: 20 mg IV every 4 weeks XRT: 60 Gy in 10 fractions

**Abbreviations:** Cy, cycle; D, day; Gy, Gray (radiation dose); IV, intravenous; mg/kg, milligram per kilogram; PD-L1, programmed death-ligand 1; XRT, radiation therapy.

A standard “3+3” study design was used for this study, and a total of 3-6 patients were enrolled per dose level in the dose-escalation phase. The maximum tolerated dose (MTD) was defined as the highest dose level with less than 2 patients with dose-limiting toxicity (DLT) out of at least 6 patients in the cohort. Once the MTD was identified, an expansion cohort allowed up to 14 patients each for additional characterization of safety and response and for correlative studies.

### Patient selection

Patients who were eligible for the study and included in this analysis had advanced or metastatic GI tumors, including pancreatic, colorectal, hepatocellular, and gastric cancers that were confirmed through histological testing and had measurable disease according to response criteria (described below). These patients had either not responded or were intolerant to standard-of-care treatment. Additionally, they had an Eastern Cooperative Oncology Group (ECOG) performance status of 0 or 1 and had adequate results on complete blood counts and tests for blood chemistry, liver function, and renal function. Patients receiving current immunosuppressive therapy or with active autoimmune disease, prior organ transplantation, or viral infections (HIV, hepatitis B virus [HBV], or hepatitis C virus [HCV]) were excluded from the trial. A full list of patient selection criteria is provided in **Table 2**.

**Table 2.** Patient eligibility criteria

Inclusion criteria To be eligible for this trial, subjects must fulfill the following criteria: 1. Subjects must be refractory to, or intolerant of, established therapy known to provide clinical benefit for their conditions, or where subjects refused existing therapies. 2. Subjects must have measurable disease (Response Evaluation Criteria in Solid Tumors [RECIST] version 1.1) or patients may have bone-metastatic disease evaluable by Prostate Cancer Working Group 2 (PCWG2) for subjects with metastatic castration-resistant prostate cancer (CRPC) or according to tumor evaluation criteria best suitable and accepted for the tumor type evaluated. 3. Age ≥18 years. 4. Eastern Cooperative Oncology Group (ECOG) performance status of 0-1. 5. Adequate hematologic function, defined as: Platelets ≥ 100 × 10^9^/L (for patients with hepatocellular carcinoma, platelets ≥ 70×10^9^/L) Hemoglobin ≥ 9 g/dL Absolute neutrophil count (ANC) ≥ 1.5 × 10^9^/L White blood cell (WBC) count ≥ 3 × 10^9^/L. 6. Adequate liver function defined as: Alanine transaminase (ALT) ≤ 2.5 x upper normal limit (ULN) (≤5 x ULN for subjects with documented metastatic disease to the liver) Aspartate aminotransferase (AST) ≤ 2.5 x ULN (≤5 x ULN for subjects with documented metastatic disease to the liver) Alkaline phosphatase < 4 x ULN Total bilirubin ≤ 1.5 x ULN (in the expansion cohort, subjects with Gilbert’s syndrome [hereditary indirect hyperbilirubinemia] must have a total bilirubin of ≤3 x ULN) Albumin ≥ 3 g/dL. 7. Renal function defined as serum creatinine ≤2 x upper limit of normal (ULN) or estimated creatinine clearance ≥ 30 mL/min as calculated using the Cockcroft-Gault formula. 8. Subject has recovered to Grade ≤ 1 by the National Cancer Institute Common Terminology Criteria for Adverse Events, version 4.03 (NCI-CTCAE v4.03)4 from the effects of recent surgery, adiotherapy, chemotherapy, hormonal therapy, or other targeted therapies, with the exception of alopecia. The exceptions for such effects are allowed lab values of ≤grade 2 specified elsewhere in these inclusion criteria. 9. Life expectancy of at least 12 weeks. 10. Negative serum pregnancy test in women of childbearing potential within 7 days of first dose of treatment, and patients of childbearing potential must agree to use effective contraception during and until 90 days after last dose. A woman of childbearing potential is defined as a premenopausal female capable of becoming pregnant. This includes women on oral, injectable, or mechanical contraception; women who are single, and women whose male sexual partners have been vasectomized or whose male sexual partners have received or are utilizing mechanical contraceptive devices. 11. Subjects must have biopsiable disease. For Arms A, B, and C, subjects must have at least two lesions amenable to biopsy and response evaluation. For Arm D, subjects should have at least three lesions amenable to biopsy, response evaluation, and radiation. Tumor lesions used for biopsy should not be lesions used as RECIST target lesions. However, if patients in Arm D do not have three separate lesions, patients will be eligible if there are two lesions, in which one is >2 cm (short axis) and can be used for both biopsy and response evaluation. 12. Subjects must give informed consent according to the rules and regulations of the individual participating sites.
Exclusion criteria Subjects with any of the following will not be eligible for the study: 1. Subjects with primary central nervous system (CNS) tumor or CNS tumor involvement. However, subjects with metastatic CNS tumors may participate in this study if the subject is: >4 weeks from prior therapy completion (including radiation and/or surgery) Clinically stable with respect to the CNS tumor at the time of study entry Not receiving steroid therapy in treating CNS tumor or CNS tumor involvement Not receiving anti-convulsive medications (that were started for brain metastases). 2. Major surgery, radiation therapy, or systemic anticancer therapy within 4 weeks of study drug administration (6 weeks for mitomycin C or nitrosoureas). Palliative radiotherapy to a limited field is allowed after consultation with the medical monitor at any time during study participation, including during screening, unless it’s clearly indicative of disease progression. 3. Subjects with prior anti-PD-1, anti-PD-L1 treatment. For Arms A and D, subjects may not have had prior 4-1BB treatment. For Arm B, subjects may not have had prior OX40 treatment. For Arm C, subjects may not have had prior 4-1BB or OX40 treatment. 4. Diagnosis or recurrence of invasive cancer other than the present cancer within 3 years (except basal or squamous cell carcinoma of the skin that has been definitively treated). 5. Clinically significant (ie, active) cardiovascular disease: cerebral vascular accident/stroke (<6 months prior to enrollment), myocardial infarction (<6 months prior to enrollment), unstable angina, congestive heart failure (≥ New York Heart Association Classification Class II), or serious cardiac arrhythmia requiring medication. 6. Active infection requiring systemic therapy. 7. Treatment with an investigational anticancer study drug within 4 weeks prior to study drug administration date. 8. Concurrent therapy with approved or investigational anticancer therapeutics. 9. Known prior severe hypersensitivity to investigational product(s) or any component in its formulations, including known severe hypersensitivity reactions to monoclonal antibodies (NCI CTCAE v4.03 Grade ≥ 3). 10. Current use of immunosuppressive medication, EXCEPT for the following: intranasal, inhaled, topical steroids, or local steroid injection (eg, intra-articular injection); systemic corticosteroids at physiologic doses ≤ 10 mg/day of prednisone or equivalent; or steroids as premedication for hypersensitivity reactions (eg, CT scan premedication). 11. Active autoimmune disease that might deteriorate when receiving an immunostimulatory agent. Patients with diabetes type I, vitiligo, psoriasis, or hypo- or hyperthyroid diseases not requiring immunosuppressive treatment are eligible. 12. Prior organ transplantation including allogenic stem-cell transplantation. 13. Known history of testing positive for HIV or known acquired immunodeficiency syndrome. 14. Hepatitis B virus (HBV) or hepatitis C virus (HCV) infection at screening (positive HBV surface antigen or HCV RNA if anti-HCV antibody screening test positive) 15. Vaccination (live attenuated virus) within 4 weeks of the first dose of avelumab and while on trial is prohibited (administration of inactivated vaccines is permitted). 16. Persisting toxicity related to prior therapy (NCI CTCAE v4.03 grade > 1); however, alopecia, sensory neuropathy grade ≤ 2, or other grade ≤ 2 not constituting a safety risk based on investigator’s judgment are acceptable. 17. Other severe acute or chronic medical conditions including colitis, inflammatory bowel disease, pneumonitis, pulmonary fibrosis, or psychiatric conditions, including recent (within the past year) or active suicidal ideation or behavior; or laboratory abnormalities that may increase the risk associated with study participation or study treatment administration or may interfere with the interpretation of study results and, in the judgment of the investigator, would make the patient inappropriate for entry into this study. 18. Medical, psychological, or social conditions that may interfere with the patient’s participation in the study or evaluation of the study results. 19. Pregnancy or lactation. 20. Men whose partner is a woman of childbearing potential (ie, biologically able to conceive) and who is not employing two forms of highly effective contraception. Highly effective contraception (eg, male condom with spermicide, diaphragm with spermicide, intrauterine device) must be used by both sexes during the study and must be continued for 90 days after the end of study treatment. A woman of childbearing potential is defined as a sexually mature woman who is not surgically sterile or who has not been naturally postmenopausal for at least 12 consecutive months (eg, who has had menses any time in the preceding 12 consecutive months). 21. A diagnosis of active scleroderma, lupus, or other rheumatologic disease which in the opinion of the treating radiation oncologist precludes safe radiation therapy. 22. Has had prior radiation therapy within the past 3 months where the high-dose area of the prior radiation would overlap with the high-dose area of the intended radiation based on the judgment of the treatment oncologist.

### Treatment plan

Dosing and treatment schedules are presented in **Table 1**. During the study, the dosing schedule of some investigational agents was revised based on available evidence and recommendations by the sponsor (Pfizer) and the investigators. Arm B (avelumab and ivuxolimab) did not have an escalation cohort because the MTD was provided by Pfizer from an ongoing study. For arm C (avelumab, ivuxolimab, and utomilumab) dose escalation was not completed because the MTD was made available from a previously conducted Pfizer protocol. In the dose-expansion cohort of Arm C, partway through the study, the initial administration of avelumab was moved from Cycle 3 Day 1 to Cycle 1 Day 15 based on recommendations by the sponsor (Pfizer) and the investigators. In each of the radiation arms (E and F), only dose level 1 was evaluated. In Arm E (avelumab, ivuxolimab, and radiation therapy), the dosing schedule was revised partway through the accrual based on recommendations by the sponsor (Pfizer) and the investigators. In schedule 1, avelumab and ivuxolimab were first administered on Cycle 1 Day 1, and 10 fractions of radiation were administered from cycle 1 day 2 to day 11. In schedule 2, ivuxolimab and avelumab were first administered on cycle 1 day 1 and cycle 1 day 15, respectively, while radiation therapy was administered from days − 14 to − 1. In Arm F (avelumab, ivuxolimab, utomilumab, and radiation therapy), only schedule 1 was tested.

In cohorts E and F, radiation therapy was prescribed to 60 Gy in 10 fractions to a designated lesion (**Tables 1 and 3**). Patients who received radiation therapy were simulated on a computed tomography (CT) scanner with customized immobilization and using motion management by breath hold or by accounting for respiratory motion (4D CT). All treatment planning was done in Raystation software, and treatment delivery was with intensity-modulated radiation therapy (IMRT) with 6 MV photons. Daily 3D image guidance was used to deliver the radiation plans.

**Table 3.** Radiation treatment schedule. Gastrointestinal cancer patients excluding biliary tract cancer patients received radiation therapy per schedule-2 version 08 and schedule-1 version 03-07.

Cohorts	Protocol version	Treatment schedule
Arm D-F escalation/expansion	Schedule-3 version 09	Avelumab starting Cy1D15; XRT starting Cy1D1-3 days, dose: 24 Gy in 3 Fx
	Schedule-2 version 08	Avelumab starting Cy1D15, XRT starting Cy1D1-14 days, dose: 60 Gy in 10 Fx
	Schedule-1 version 03-07	Avelumab starting Cy1D1, XRT starting Cy1D2-11 days, dose: 60 Gy in 10 Fx

**Abbreviations:** Cy, cycle; D, day; Fx, fractions; Gy, Gray (radiation dose); IV, intravenous; XRT, radiation therapy.

### Study objectives and endpoints

The primary objectives of this study were to assess safety, tolerability, and DLTs of different treatment combinations of avelumab with checkpoint agonist(s) with or without radiation in patients with advanced or metastatic GI tumors, in order to estimate the maximum tolerated dose (MTD) and select the recommended phase 2 dose (RP2D). Safety and tolerability were assessed by monitoring the frequency, duration, and severity of adverse events (AEs).

The secondary objectives of this study were to evaluate the efficacy of the treatment combinations in patients with advanced or metastatic GI tumors by assessing objective response rate per Response Evaluation Criteria in Solid Tumors (RECIST) version 1.1 and immune-related RECIST (irRECIST).^1,2^ Additionally, progression-free survival (PFS) and overall survival (OS) were assessed using the Kaplan-Meier method.

### Response assessment

Tumor imaging was performed at baseline and after 2 cycles or 8 weeks of treatment. Three versions of the RECIST criteria were used. irRECIST was used to direct clinical management and report the efficacy of the regimens.^1,2^ RECIST v 1.1 was used as an additional method to describe radiological responses, in order to provide insights into the unique patterns of response associated with immunotherapeutic agents, such as pseudoprogression and mixed responses. For arms E and F, mRECIST, a modified version of RECIST 1.1, was used to evaluate radiated target lesions.^3^ Patients were categorized as having immune-related complete response (irCR), partial response (irPR), stable disease (irSD), or progressive disease (irPD). Objective response, defined as irCR or irPR, required confirmation with a follow-up scan, with the scans at least 4 weeks apart. While not mandatory, a confirmatory follow-up scan was also suggested to confirm progressive disease.

### Safety assessment

Toxicity was graded according to National Cancer Institute Common Terminology Criteria for Adverse Events (CTCAE) version 4.03. Treatment-related AEs (AEs considered caused by the treatment) and treatment-emergent AEs (AEs that occurred during treatment but were not considered caused by the treatment) were tabulated. DLTs were defined as AEs that were related to treatment and occurred during the first 2 cycles or 8 weeks of treatment.

## Statistical analysis

We used descriptive statistics to summarize patient characteristics. For categorical variables, summary tabulations of the number and percentage of subjects within each category of the parameter are presented. For continuous variables, the number of subjects, median, minimum, and maximum values were calculated. The safety analysis set included all enrolled patients who received at least one dose of each study medication in the assigned treatment combination. The best overall response was defined as the best response using irRECIST during treatment. Disease control rate was defined as the percentage of patients with irCR, irPR, or at least 6 months of irSD. A waterfall plot was used to illustrate the maximum percentage of change in tumor measurements from baseline per irRECIST. A swimmer plot was generated to visualize the duration of treatment and the events of disease progression (per irRECIST), best overall response of stable disease (per irRECIST), death, and censoring time of each patient. Progression-free survival was defined as the time from the cycle 1 start date until the earliest date of disease progression, as determined by investigator evaluation of objective radiographic disease assessments per irRECIST, or death due to any cause, if occurring sooner than progression. Overall survival was defined as the time from the cycle 1 start date until death due to any cause. Time-to-event data were summarized using Kaplan–Meier methodology with associated two-sided 95% confidence intervals (95% CIs). The data were analyzed using SAS 9.4 statistical software.

## Ethics, institutional review board approval, and consent

The protocol was approved by the Institutional Review Board at The University of Texas MD Anderson Cancer Center. This study was conducted in accordance with current FDA regulations, Good Clinical Practice, the International Council for Harmonization of Technical Requirements for Pharmaceuticals for Human Use guidelines, the ethical principles stated in the Declaration of Helsinki, and all local ethical and legal requirements. All participants in the study provided written informed consent prior to enrollment.

## Drug Information

**Table:** 

**Generic/working name**	Avelumab, ivuxolimab, utomilumab
**Company name**	MSB-0010718C, PF-04518600, PF 05082566
**Drug type**	PD-L1 inhibitor, OX40 agonist, 4-1BB agonist
Drug Class	Immunotherapy
Dose	Avelumab: 10 mg/kg Ivuxolimab: 0.3 mg/kg Utomilumab: 20 mg
**Unit**	Variable (see above)
**Route**	IV
Schedule of administration	Variable

## Drug Information (multi-arm trial)

**Table:** 

Arm	Arm 1 (*n* = 8)	Arm 2 (*n* = 3)	Arm 3 (*n* = 16)	Arm 4 (*n* = 1)	Arm 5 (*n* = 1)	Arm 6 (*n* = 2)
**Generic/working name**	B expansion	C escalation	C expansion	E Schedule 1	E schedule 2	F
**Company name drug type**	Avelumab + Ivuxolimab	Avelumab + Ivuxolimab + Utomilumab	Avelumab + Ivuxolimab + Utomilumab	Avelumab + Ivuxolimab + radiation therapy	Avelumab + Ivuxolimab + radiation therapy	Avelumab + Ivuxolimab + Utomilumab + radiation therapy
Drug class	PD-L1 inhibitor, OX40 agonist	PD-L1 inhibitor, OX40 agonist, 4-1BB agonist	PD-L1 inhibitor, OX40 agonist, 4-1BB agonist	PD-L1 inhibitor, OX40 agonist	PD-L1 inhibitor, OX40 agonist	PD-L1 inhibitor, OX40 agonist, 4-1BB agonist
Dose	Avelumab: 10 mg/kg Ivuxolimab: 0.3 mg/kg	Avelumab: 10 mg/kg Ivuxolimab: 0.3 mg/kg Utomilumab: 20 mg	Avelumab: 10 mg/kg Ivuxolimab: 0.3 mg/kg Utomilumab: 20 mg	Avelumab: 10 mg/kg Ivuxolimab: 0.3 mg/kg; RT: 60 Gy in 10 fractions	Avelumab: 10 mg/kg Ivuxolimab: 0.3 mg/kg; RT: 60 Gy in 10 fractions	Avelumab: 10 mg/kg Ivuxolimab: 0.3 mg/kg Utomilumab: 20 mg; RT: 60 Gy in 10 fractions
**Unit**	As above	As above	As above	As above	As above	As above
**Route**	IV	IV	IV	IV	IV	IV
Schedule of Administration	Both every 2 weeks; Ivuxolimab: starting cycle 1 day 1, Avelumab: initially started cycle 3 day 1; partway revised to start cycle 1 day 15	Avelumab + Ivuxolimab every 2 weeks; Utomilumab every 4 weeks. Avelumab, ivuxolimab, and utomilumab all started on cycle 1 day 1	Avelumab + Ivuxolimab every 2 weeks; Utomilumab every 4 weeks. Ivuxolimab and utomilumab: starting cycle 1 day 1, Avelumab: initially started cycle 3 day 1; partway revised to start cycle 1 day 15	Avelumab + Ivuxolimab every 2 weeks, both starting cycle 1 day 1, radiation therapy: starting cycle 1 day 2 (through cycle 1 day 11)	Avelumab + Ivuxolimab every every 2 weeks. Ivuxolimab: starting cycle 1 day 1, Avelumab: starting cycle 1 day 15, radiation therapy starting from day −14 through day −1	Avelumab + Ivuxolimab every 2 weeks; Utomilumab every 4 weeks. Avelumab, ivuxolimab, and utomilumab: all starting cycle 1 day 1, radtiation therapy from cycle 1 day 2 to cycle 1 day 11)

## Patient Characteristics

**Table:** 

	
Number of patients, male	**22**
Number of patients, female	**9**
**Stage**	IV
Age: Median (range)	63 (36-74)
Number of prior systemic therapies: median (range)	2 (0-7)
Performance status: ECOG	0:4 1:27 2:0 3:0 4:0
Cancer types or histologic subtypes	Pancreatic: 21 Colon: 5 Rectal: 3 Stomach: 1 Liver: 1

## Primary Assessment Method

**Table:** 

Title	Safety and Efficacy for all arms
Number of patients screened	
Number of patients enrolled	31
Number of patients evaluable for toxicity	31
Number of patients evaluated for efficacy	24
Evaluation method	irRECIST
Response assessment, CR	0 (0%)
Response assessment, PR	0 (0%)
Response assessment, SD	9 (37.5%)
Response assessment, PD^*^	15 (62.5%)

* Fourteen (58.3%) patients experienced immune-related progression disease (irPD), while 1 (4.2%) patient had clinical progression without radiological confirmation.

**Table:** 

(Median) Duration assessments	#	Day/week/month?	CI
PFS	2	Months	1.8-3.4
TTP			
OS	5.6	Months	3.6-8.4
Response duration			
Duration of treatment			

## Outcome notes

In this subgroup analysis in patients with advanced GI malignancies, 31 patients—pancreatic (*n* = 21), colorectal (*n* = 8), hepatocellular (*n* = 1), and gastric (*n* = 1) cancers—were enrolled in the study between October 27, 2017, and September 16, 2019. Among these patients, 27 were treated in arms B and C, and only 4 patients were treated in arms E and F (with radiation). Patient demographic characteristics are summarized in Table 4. The median age of patients was 63 years (range 36-74 years), 71% of patients were male, and 68% were white.

**Table 4.** Patient characteristics.

Baseline characteristic	*N*
Age, years	
Median	63
Range	36-74
Sex	
Male	22 (71)
Female	9 (29)
Race	
White	21 (68)
Black	6 (19)
Asian	2 (7)
Other	2 (7)
ECOG status	
0	4 (13)
1	27 (87)
Tumor type	
Pancreatic	21(68)
Colon	5(16)
Rectal	3 (10)
Stomach	1 (3)
Liver	1 (3)
Number of prior lines of treatment	
Median	2
Range	0-7
Cohort	
Arm B Expansion, avelumab starting from C1D15	7 (23)
Arm B Expansion, avelumab starting from C3D1	1 (3)
Arm C Escalation	3 (10)
Arm C Expansion, avelumab starting from C1D15	8 (26)
Arm C Expansion, avelumab starting from C3D1	8 (26)
Arm E Escalation, Schedule 1, Dose Level 1	1 (3)
Arm E Escalation, Schedule 2, Dose Level 1	1 (3)
Arm F Escalation, Schedule 1, Dose Level 1	2 (7)

Abbreviations: C, cycle; D, day; ECOG, Eastern Cooperative Oncology Group; N, number of patients.

### Safety

Thirty-one patients were evaluable for safety. Treatment-related AEs for the 31 patients are presented in Table 5. The most common were chills (4 patients, 13%), diarrhea (3 patients, 10%), colitis (3 patients, 10%), fatigue (2 patients, 6%), and fever (2 patients, 6%). Grade 3 diarrhea and grade 3 colitis were observed in 3 patients (10%) each; all other treatment-related AEs were either grade 1 or 2.

**Table 5.** Treatment-related adverse events.

Adverse event*	All grades *N* (%)	Grade 1 or 2 *N* (%)	Grade 3 *N* (%)
Chills	4 (13)	4 (13)a, b, c, d	0 (0)
Diarrhea	4 (13)	1 (3)^b^	3 (10)b, d, d
Colitis	3 (10)	0 (0)	3 (10)a,d,d
Fatigue	2 (6)	2 (6)b, b	0 (0)
Fever	2 (6)	2 (6)a, d	0 (0)
Skin rash	1 (3)	1 (3)a	0 (0)
Hypomagnesemia	1 (3)	1 (3)c	0 (0)
Pruritus	1 (3)	1 (3)d	0 (0)
Serum lipase increased	1 (3)	1 (3)b	0 (0)
Dysgeusia	1 (3)	1 (3)b	0 (0)
Infusion-related reaction	1 (3)	1 (3)b	0 (0)
Lower gastrointestinal hemorrhage	1 (3)	1 (3)d	0 (0)

Abbreviation**s:***N*, number of patients.

*There was no grade 4 or grade 5 adverse events related to treatment.

aArm B. bArm C. cArm E. dArm F.

Treatment-emergent AEs were also most commonly grade 1 or 2 and included fatigue (35.5%), dyspnea (22.6%), anorexia (22.6%), anemia (19.4%), diarrhea (16.1%), abdominal pain (16.1%), chills (16.1%), and increased alkaline phosphatase level (16.1%). A complete list of treatment-emergent AEs is presented in Table 6. One patient (3.2%) had grade 4 lymphopenia, and 1 patient (3.2%) died of a lung infection (grade 5). These grade 4 and 5 AEs were determined not to be related to the study treatment.

**Table 6.** Treatment-emergent adverse events among the 31 patients.

Adverse event	All grades *N* (%)	Grade 1 or 2 *N* (%)	Grade 3 *N* (%)	Grade 4 *N* (%)	Grade 5 *N* (%)
Fatigue	11 (35.5)	10 (32.3)	1 (3.2)	0 (0)	0 (0)
Dyspnea	7 (22.6)	3 (9.7)	4 (12.9)	0 (0)	0 (0)
Anorexia	7 (22.6)	7 (22.6)	0 (0)	0 (0)	0 (0)
Anemia	6 (19.4)	6 (19.4)	0 (0)	0 (0)	0 (0)
Diarrhea	5 (16.1)	3 (9.7)	2 (6.5)	0 (0)	0 (0)
Abdominal pain	5 (16.1)	3 (9.7)	2 (6.5)	0 (0)	0 (0)
Chills	5 (16.1)	5 (16.1)	0 (0)	0 (0)	0 (0)
Alkaline phosphatase increased	5 (16.1)	4 (12.9)	1 (3.2)	0 (0)	0 (0)
Hyponatremia	4 (12.9)	1 (3.2)	3 (9.7)	0 (0)	0 (0)
Fever	4 (12.9)	4 (12.9)	0 (0)	0 (0)	0 (0)
Constipation	4 (12.9)	3 (9.7)	1 (3.2)	0 (0)	0 (0)
Skin rash	4 (12.9)	3 (9.7)	1 (3.2)	0 (0)	0 (0)
Thromboembolic event	4 (12.9)	1 (3.2)	3 (9.7)	0 (0)	0 (0)
AST increased	3 (9.7)	2 (6.5)	1 (3.2)	0 (0)	0 (0)
Back pain	3 (9.7)	3 (9.7)	0 (0)	0 (0)	0 (0)
Colitis	3 (9.7)	0 (0)	3 (9.7)	0 (0)	0 (0)
Insomnia	3 (9.7)	3 (9.7)	0 (0)	0 (0)	0 (0)
Lung infection	3 (9.7)	1 (3.2)	1 (3.2)	0 (0)	1 (3.2)
Hypomagnesemia	2 (6.5)	2 (6.5)	0 (0)	0 (0)	0 (0)
Hypokalemia	2 (6.5)	1 (3.2)	1 (3.2)	0 (0)	0 (0)
Cough	2 (6.5)	2 (6.5)	0 (0)	0 (0)	0 (0)
Hypophosphatemia	2 (6.5)	2 (6.5)	0 (0)	0 (0)	0 (0)
ALT increased	2 (6.5)	1 (3.2)	1 (3.2)	0 (0)	0 (0)
Ascites	2 (6.5)	1 (3.2)	1 (3.2)	0 (0)	0 (0)
Limb edema	2 (6.5)	2 (6.5)	0 (0)	0 (0)	0 (0)
White blood cells decreased	2 (6.5)	2 (6.5)	0 (0)	0 (0)	0 (0)
Small intestinal obstruction	2 (6.5)	0 (0)	2 (6.5)	0 (0)	0 (0)
Serum lipase increased	2 (6.5)	2 (6.5)	0 (0)	0 (0)	0 (0)
Creatinine increased	2 (6.5)	2 (6.5)	0 (0)	0 (0)	0 (0)
Nausea	2 (6.5)	2 (6.5)	0 (0)	0 (0)	0 (0)
Pruritus	2 (6.5)	2 (6.5)	0 (0)	0 (0)	0 (0)
Upper respiratory infection	2 (6.5)	2 (6.5)	0 (0)	0 (0)	0 (0)
Tumor pain	2 (6.5)	2 (6.5)	0 (0)	0 (0)	0 (0)
Weight loss	1 (3.2)	1 (3.2)	0 (0)	0 (0)	0 (0)
Lymphopenia	1 (3.2)	0 (0)	0 (0)	1 (3.2)	0 (0)
Thrombocytopenia	1 (3.2)	1 (3.2)	0 (0)	0 (0)	0 (0)
Hyperbilirubinemia	1 (3.2)	1 (3.2)	0 (0)	0 (0)	0 (0)
Hydronephrosis	1 (3.2)	1 (3.2)	0 (0)	0 (0)	0 (0)
Epigastric pain	1 (3.2)	1 (3.2)	0 (0)	0 (0)	0 (0)
Testicular pain	1 (3.2)	1 (3.2)	0 (0)	0 (0)	0 (0)
Bone pain	1 (3.2)	1 (3.2)	0 (0)	0 (0)	0 (0)
Pain in extremity	1 (3.2)	1 (3.2)	0 (0)	0 (0)	0 (0)
Hypocalcemia	1 (3.2)	1 (3.2)	0 (0)	0 (0)	0 (0)
Adjustment disorder with depressive mood	1 (3.2)	1 (3.2)	0 (0)	0 (0)	0 (0)
Serum amylase increased	1 (3.2)	1 (3.2)	0 (0)	0 (0)	0 (0)
Dry skin	1 (3.2)	1 (3.2)	0 (0)	0 (0)	0 (0)
Hyperkalemia	1 (3.2)	1 (3.2)	0 (0)	0 (0)	0 (0)
Vomiting	1 (3.2)	1 (3.2)	0 (0)	0 (0)	0 (0)
Dysgeusia	1 (3.2)	1 (3.2)	0 (0)	0 (0)	0 (0)
Pneumothorax	1 (3.2)	1 (3.2)	0 (0)	0 (0)	0 (0)
APTT prolonged	1 (3.2)	1 (3.2)	0 (0)	0 (0)	0 (0)
CPK increased	1 (3.2)	0 (0)	1 (3.2)	0 (0)	0 (0)
Skin infection	1 (3.2)	1 (3.2)	0 (0)	0 (0)	0 (0)
Colostomy irritation	1 (3.2)	1 (3.2)	0 (0)	0 (0)	0 (0)
Urinary retention	1 (3.2)	1 (3.2)	0 (0)	0 (0)	0 (0)
Infusion-related reaction	1 (3.2)	1 (3.2)	0 (0)	0 (0)	0 (0)
Photosensitivity	1 (3.2)	1 (3.2)	0 (0)	0 (0)	0 (0)
INR increased	1 (3.2)	1 (3.2)	0 (0)	0 (0)	0 (0)
Pleural effusion	1 (3.2)	1 (3.2)	0 (0)	0 (0)	0 (0)
Cholecystitis	1 (3.2)	0 (0)	1 (3.2)	0 (0)	0 (0)
Agitation	1 (3.2)	1 (3.2)	0 (0)	0 (0)	0 (0)
Lower gastrointestinal hemorrhage	1 (3.2)	1 (3.2)	0 (0)	0 (0)	0 (0)
Generalized muscle weakness	1 (3.2)	1 (3.2)	0 (0)	0 (0)	0 (0)
Depression	1 (3.2)	1 (3.2)	0 (0)	0 (0)	0 (0)
Lymphedema	1 (3.2)	1 (3.2)	0 (0)	0 (0)	0 (0)
Hallucinations	1 (3.2)	1 (3.2)	0 (0)	0 (0)	0 (0)
Gait disturbance	1 (3.2)	1 (3.2)	0 (0)	0 (0)	0 (0)
Hyperglycemia	1 (3.2)	0 (0)	1 (3.2)	0 (0)	0 (0)
Nasal congestion	1 (3.2)	1 (3.2)	0 (0)	0 (0)	0 (0)
Gastroesophageal reflux disease	1 (3.2)	1 (3.2)	0 (0)	0 (0)	0 (0)
Neutropenia	1 (3.2)	1 (3.2)	0 (0)	0 (0)	0 (0)
Urinary frequency	1 (3.2)	1 (3.2)	0 (0)	0 (0)	0 (0)
Urinary tract infection	1 (3.2)	1 (3.2)	0 (0)	0 (0)	0 (0)
Hypertension	1 (3.2)	1 (3.2)	0 (0)	0 (0)	0 (0)
Abdominal distension	1 (3.2)	1 (3.2)	0 (0)	0 (0)	0 (0)

Abbreviation: ALT, alanine transaminase; APTT, activated partial thromboplastin time; AST, aspartate aminotransferase; CPK, creatine phosphokinase; INR, international normalized ratio; N, number of patients.

### Efficacy

Of the 31 total patients with advanced GI malignancies, 7 were non-evaluable for response assessment. Five patients came off the treatment prior to receiving at least one dose of all the drugs in the combination. One patient withdrew consent prior to receiving at least one dose of all the drugs in the combination, and another patient withdrew consent prior to first restaging.

**Figure 1.**
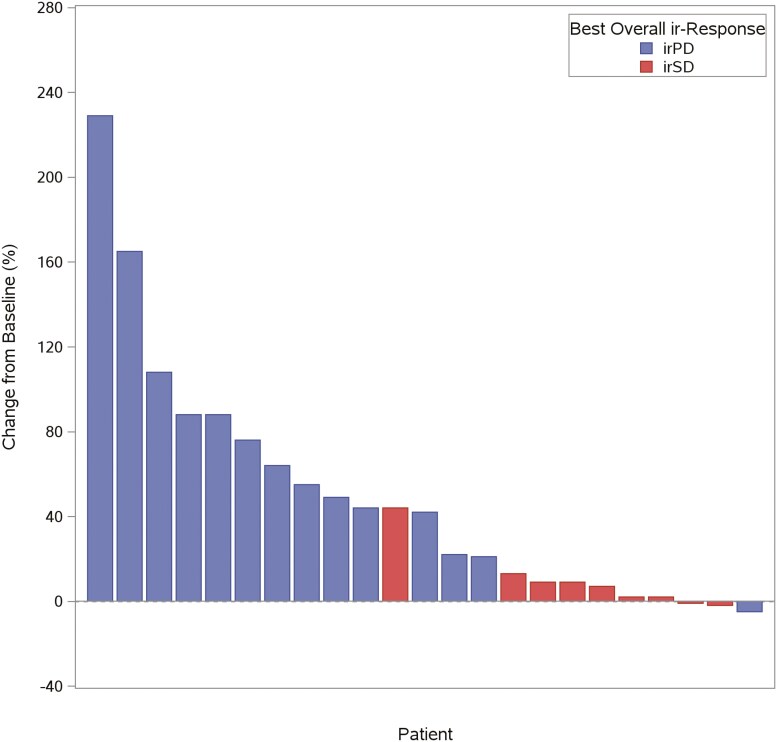
Waterfall plot showing best response change from baseline (% change) (by irRECIST) for the 24 patients evaluable for response. Each bar in the figure represents 1 patient in the study.

**Figure 2.**
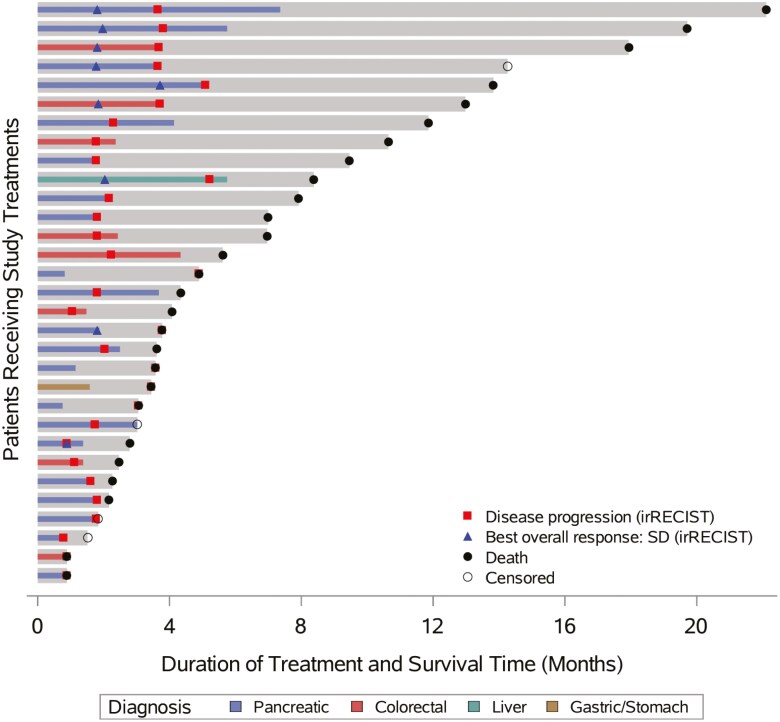
Swimmer plot censored by disease group based on irRECIST criteria. Each gray bar represents 1 patient in the study. The colored lines represent the time on treatment. Colors indicate the type of malignancy.

Of the 24 patients evaluable for response, using irRECIST for assessment, no patients had irCR or irPR, and 9 had irSD, for a disease control rate of 37.5%. Fourteen (58.3%) patients had irPD, and 1 (4.2%) patient had clinical progression without radiological confirmation. Patients’ best responses in relation to baseline are presented in **Figure 1**. Additional data are presented in **Figure 2**. Response evaluation was also done by RECIST 1.1 to identify patients with atypical response patterns associated with immunotherapy. Only 1 patient had a different result with the two methods; this patient had irPD as best response by RECIST 1.1 and irSD as best response by irRECIST. However, the patient came off treatment soon after due to clinical progression without radiological confirmation.

**Figure 3.**
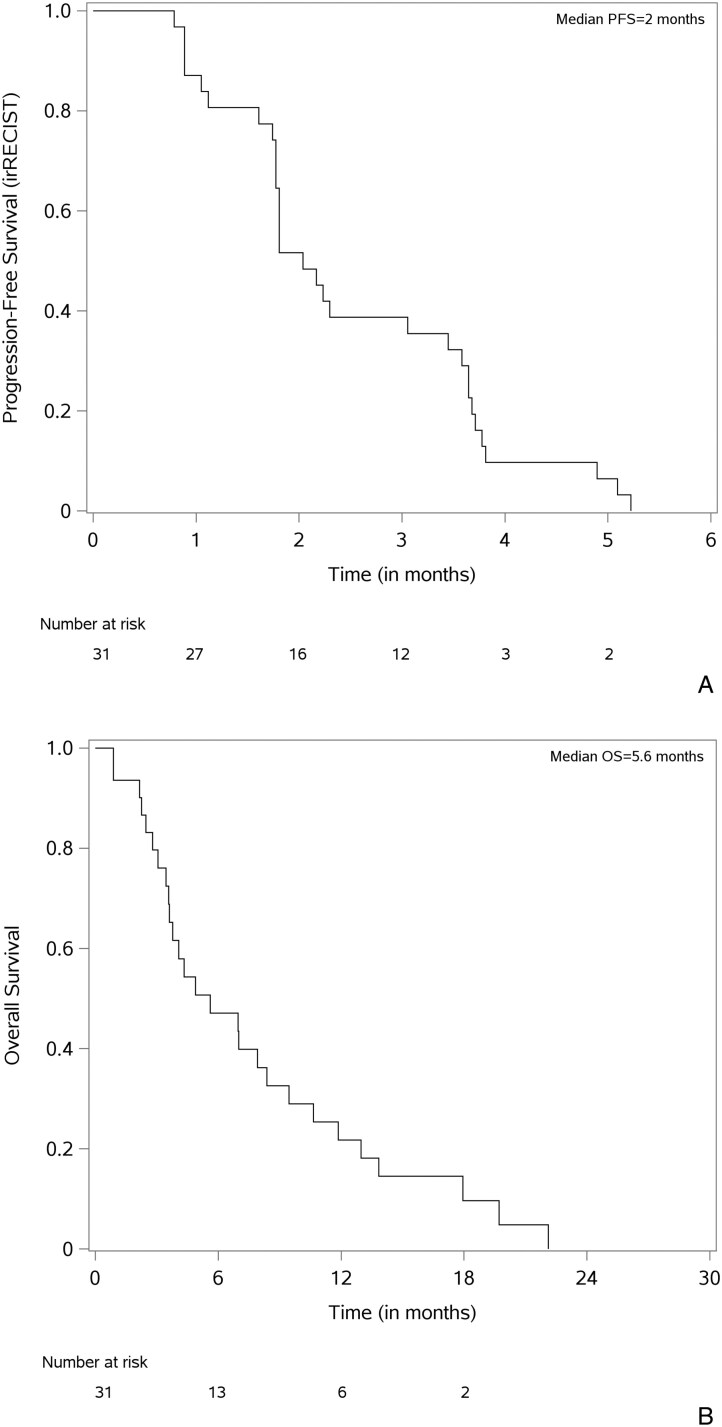
Kaplan-Meier plots. (A) Progression-free survival (PFS), with progression defined per irRECIST guidelines. (B) Overall survival (OS).

The median PFS was 2.0 months (95% CI, 1.8-3.4 months), and the median OS was 5.6 months (95% CI, 3.6-8.4 months) (**Figure 3A and B**).

## Assessment, Analysis, and Discussion

**Table:** 

Completion	Study completed
**Investigator’s assessment**	Level of activity did not meet planned end point

This is a phase 1/2 multicohort trial of anti-PD-L1 checkpoint inhibitor avelumab in combination with co-stimulatory checkpoint agonist(s) and radiation therapy in patients with advanced GI malignancies. Apart from the anticipated AEs associated with anti-PD-L1 treatment, no added safety concerns were observed. Overall, the treatment combination produced mainly grade 1-2 AEs with no grade 4 or 5 AEs attributable to the study drugs. Exclusively 3 patients (9.7%) experienced grade 3 diarrhea and colitis in the study.

The clinical benefit of the combination treatments was limited to a best response of irSD in 9 patients (37.5%) per irRECIST, and 8 of these 9 patients were treated in arms B and C with combination immunotherapy (no radiation therapy). Thus, despite encouraging findings from preclinical *in vivo* studies, an interpretation about the effects of radiation therapy in this clincial trial is not possible due to the small number of patients treated in arms E and F.^4–9^ Among the patients with stable disease, 6 patients had pancreatic cancer, 2 had colorectal cancer, and 1 had hepatocellular carcinoma. Prior clinical trials have shown limited clinical benefit to single-agent anti-PD-L1 therapy in pancreatic and colorectal cancer.^10,11^ Preclinical animal studies combining co-stimulatory checkpoint agonists with checkpoint inhibitors have demonstrated their synergistic role in controlling tumor growth, reducing T-cell exhaustion and Treg populations, and promoting T-cell persistence and memory.^12–17^ Despite promising results in the preclinical studies, the addition of co-stimulatory checkpoint agonists OX40 and 4-1BB did not produce meaningful improvement in our study. A prior first-in-human phase 1 clinical trial combining ivuxolimab (OX40) and utomilumab (4-1BB) agonistic antibodies resulted in stable disease in 31.6% of patients and a disease control rate of 35.1%; however, the study was limited mainly to non-GI tumors such as non-small cell lung cancers, head and neck cancers, and melanoma, which tend to be more responsive to immunotherapies.^18^

Our study also explored the role of sequential treatment by stimulating the T cells with OX40 agonist ivuxolimab, followed by anti-PD-L1 treatment with avelumab (arms B and C), which has demonstrated an additive effect in prior preclinical mouse mammary cancer model.^19^ However, changes in the dosing schedule midway through our clinical trial precluded us from making significant conclusions.

Our study has certain limitations. A heterogeneous group of GI malignancies were included in this trial, although most patients had either pancreatic or colorectal cancers. The small number of patients within each group, the multiple treatment arms, and changes in dosing schedule during the study present challenges to drawing conclusions from the available data. However, ongoing correlative studies could inform the design of future studies in this patient population. An understanding of the immune correlates, T-cell agonistic antibody expression, and the intricacies in the tumor microenvironment in pancreatic and colorectal cancers will provide insight into strategies for combining checkpoint inhibitors with a growing number of co-stimulatory checkpoint agonists, with or without concurrent targeted therapy, chemotherapy, and radiation therapy.

## Data Availability

The datasets used and/or analyzed during the current study are available from the corresponding author upon request and approval from study sponsor according to available guidelines at time of request.
